# Toward a genomic understanding of the tree of life

**DOI:** 10.1093/molbev/msag043

**Published:** 2026-02-13

**Authors:** Anja Spang, Davide Pisani

**Affiliations:** Department of Marine Microbiology and Biogeochemistry, NIOZ, Royal Netherlands Institute for Sea Research, AB Den Burg, The Netherlands; Department of Evolutionary and Population Biology, Institute for Biodiversity and Ecosystem Dynamics (IBED), University of Amsterdam, Amsterdam, The Netherlands; Palaeobiology Research Group, School of Biological Sciences, University of Bristol, Bristol, UK

**Keywords:** phylogenomics, metazoa, eukaryota, tree of life

**Figure 1:**
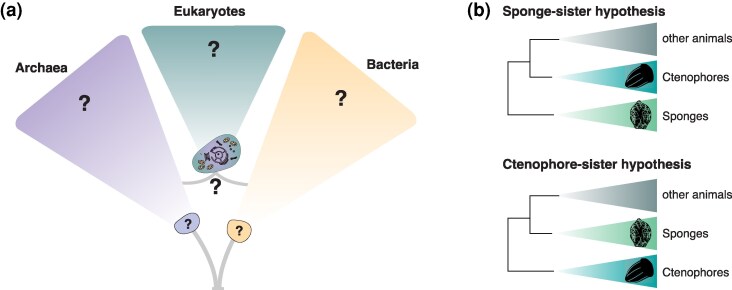
Schematic tree of life depicting (a) the origin of eukaryotes through a symbiosis between archaea and bacteria and (b) the debate surrounding the early evolution of animals. The animal silouettes were released under license cc0 1.0 Universal Public domain dedication. The sponge Oopsacas minuta is by Darrin Shultz – https://www.phylopic.org/images/36309207–addf. Beroe forskalii is by MBARI and FathomVerse – https://www.phylopic.org/images/f1351e60-4ed7-4ad8-aab8-d6e1858ab112/beroe-forskalii

The history of phylogenetics has entered a crucial time as limited availability of genomic data is quickly becoming a thing of the past (eg Lewin et al. 2022). Accordingly, Molecular Biology and Evolution has taken the decision to renew its effort to publish high-quality phylogenomic research and invites submissions to a new Call of Paper on Major Transitions of Life, spanning all areas of molecular phylogenetic research (both theoretical and applied) across the full tree of life (Fig. 1).

Molecular phylogenetics is a relatively young area of research that can be traced back to the work by Linus Pauling and Margaret Dayhoff in the 1960s (Zuckerkandl and Pauling 1965; Eck and Dayhoff 1966). Since its foundation in 1983, Molecular Biology and Evolution has been a reference for the phylogenetic community. Tajima and Nei's distances (Tajima and Nei 1984) were described in its first volume, and the Neighbor Joining algorithm (Saitou and Nei 1987) was published in its fourth volume. Other examples of groundbreaking papers that appeared in Molecular Biology and Evolution include the mathematical foundations of Bayesian phylogenetics (Rannala and Yang 1996; Yang and Rannala 1997), posterior predictive tests of model adequacy (Bollback 2002), and mixture models of amino acid substitution (Lartillot and Philippe 2004). See Russo et al. (2024) for an in-depth review.

In 1995, the first complete genome, for the bacterium *Haemophilus influenzae*, was sequenced (Fleischmann et al. 1995). Since then, we witnessed a series of revolutions in genome sequencing technologies that opened the way to the development of new areas of scientific research, including phylogenomics, where datasets assembled from genome-scale screens are used to infer phylogenetic relationships. Molecular Biology and Evolution has long played a key role in this area, publishing, for example, early phylogenomic support for Ecdysozoa and the “new animal phylogeny” (Philippe et al. 2005) and early genome-scale evidence supporting a two-Domain tree of life and the symbiotic roots of the eukaryotic genome (Pisani et al. 2007). However, the tree of life is vast (Fig. 1a), and the placement of many taxa remains ambiguous (eg Steenwyk et al. 2023).

We now have access to a vast amount of data from across our biosphere, for example, Lewin et al. (2022), that provide a rich source for addressing key unresolved questions about the phylogenetic relationship and evolution of cellular life (Fig. 1) as well as their viruses. However, access to more data has frequently proved insufficient to resolve difficult phylogenetic questions (Steenwyk et al. 2023). Results of phylogenetic analyses are inherently data, method, and model dependent and the availability of genomic information coupled with the tremendous progress in computational approaches of the last two decades also led to the questioning of many results that by the turn of the millennium would have been considered unshakable. For example, the monophyly of the Deuterostomia, the animal lineage traditionally assumed to join chordates, emichordates, and echinoderms, has been questioned by analyses of genomic data grounded on novel amino acid substitution models that better account for the across-site compositional heterogeneity of multiple sequence alignments (Philippe et al. 2019; Kapli et al. 2021; Serra Silva et al. 2025). Similarly, at the root of the animal tree, instability in the placement of the comb jellies (phylum Ctenophora) with reference to the sponges, which were traditionally considered the sister of all the other animals, fueled two decades of uninterrupted debate, from the publication of Dunn et al. (2008) to that of Steenwyk and King (2025), Fig. 1b. While it is impossible to do due justice to the bewildering diversity of complex phylogenetic problems that have been revisited in a phylogenomic framework, we will focus here on two iconic cases—the debate on the root of the animal phylogeny, and that of the phylogenetic relationships between Eukaryotes, Archaea, and Bacteria (Fig. 1). Both debates are highly instructive of the challenges we are facing and the direction the community is moving to.

Animals have long played a key role as models for the study of evolution—Darwin himself wondered about the abrupt appearance of (mostly animal) fossils in rocks from the “Cambrian system” (Darwin 1859). For the entirety of the 20th century, it was deemed unquestionable that the sponges (Phylum Porifera) represented the sister lineage to all the other animals (Fig. 1b). Everything changed when the phylogeny of the animal phyla was revisited by Dunn et al. (2008), using a taxonomically well-sampled EST (expressed sequence tags) dataset. While comparatively small to current standards, the tree of Dunn et al. (2008) was based on a dataset that at the time of its publication was undoubtedly of genomic scale: 150 genes and 77 species. While Dunn et al. (2008) confirmed many previous results, such as the monophyly of Ecdysozoa—the clade of molting animals including, among others, the nematodes and the arthropods (Aguinaldo et al. 1997), they did not find the sponges as the sister of all the other animals. Instead, Dunn et al. (2008) recovered the comb jellies (Phylum Ctenophora) to represent the first animal phylum to split off from the metazoan tree. This ignited a debate that lasted for two decades and does not seem to be any closer to an end now than it was when Philippe et al. (2009), using a different dataset, reinstated the sponges as the sister of all the other animal phyla. In retrospect, we now know that the Philippe et al. (2009) study was just the first in a series of investigations that kept flip-flopping the sponges and the ctenophores as the most likely sister of all the other animals, bringing the whole community on a major rollercoaster ride. What has been particularly fascinating in this debate is that every newly generated dataset could be shown to have the potential to support both tree topologies, and that support for one tree over the other was entirely dependent on how taxa were sampled and the data modeled, for example, Ryan et al. (2013) and Whelan et al. (2015) versus Pisani et al. (2015). This instability fueled a major debate but also inspired the development and application of a vast range of cutting-edge approaches, which feature prominently in modern phylogenomic research. Examples include the development of the CAT-Posterior Mean Sites Frequency (CAT-PMSF) method (Szantho et al. 2023), new posterior predictive tests of model adequacy (Feuda et al. 2017), better performing Bayesian, relative model fit tests (Lartillot 2023), the study of the relative strengths and weaknesses of different substitution models (Whelan and Halanych 2017; Wang et al. 2019; Li et al. 2021; Banos et al. 2024), and of amino-acid recoding strategies (Hernandez and Ryan 2021; Li et al. 2021; Giacomelli et al. 2022; Foster et al. 2023). The introduction of new genomic datatypes, for example, gene content data (Ryan et al. 2013) and chromosome fusions (Simakov et al. 2022; Schultz et al. 2023), and subsequent improvements in methods for their analyses (Pisani et al. 2015; Pett et al. 2019; Juravel et al. 2023; Copley 2025) contributed significantly to the debate. For instance, Schultz et al. (2023) recently revitalized a possible placement of Ctenophora at the root of the animal phylogeny using chromosome fusion data, just for Copley (2025) to show that Schultz et al. (2023) overstated the significance of their conclusions that was based on inappropriate randomization tests that inflated significance. More importantly, chromosome fusions lack power when used to compare the genomes of animals and their outgroups, where homology of ancestral linkage groups (ALGs) is based on a very small number of genes, making it impossible to test whether the ALGs represent homology or convergence (Copley 2025). The difficulty of solving the relationships at the root of the animal phylogeny is further illustrated by the debate that ensued following the latest major study published on this topic, that of Steenwyk and King (2025). These authors developed a new approach to try identifying genes that should be used to test phylogenetic hypotheses, which they referred to as “integrative phylogenomics.” Using this approach, they found new support for sponges as the sister of all the other animals, just for Dunn et al. (2026) to demonstrate that their results were undisputably affected by errors. While this led to the retraction of the paper of Steenwyk and King (2025), Steenwyk and King (2026) reply to Dunn et al. (2026) demonstrated that there is integrity in the community, as well as the self-correcting power of the scientific approach. At this stage, there is only one aspect of the debate on the relationships of the early animals that, in our opinion, is certain, and it is that the discussion around this topic is far from closed. As frustrating as this might seem, this is also good news because digesting the latest papers (Schultz et al. 2023; Steenwyk and King 2025) and the criticisms of these papers (Copley 2025; Dunn et al. 2026) will further sharpen our collective mind, leading to new advances that will further enrich our research community.

A second fascinating debate centers around the origin of complex eukaryotic cells—a key transition in the evolution of life on Earth. Environmental genomic data and improvements in phylogenomic approaches have provided new avenues to tackle this decade-long mystery. Specifically, metagenomic sequencing approaches have led to the discovery of a previously unknown archaeal lineage, which was referred to as the Asgard archaea (Spang et al. 2015; Zaremba-Niedzwiedzka et al. 2017), now officially referred to as Asgardarchaeota (Tamarit et al. 2024) and Prometheoarchaeota (Imachi et al. 2024), and whose members encode various so-called eukaryotic signature proteins (ESPs), see Vosseberg et al. (2024). These ESPs seem to encode distant homologs of eukaryotic proteins that play key roles in eukaryotic cell biology including their cytoskeletons, trafficking, and signaling machinery. Phylogenetic analyses implementing complex models of evolution have recovered the branch leading to eukaryotes (the nuclear ancestry; Donoghue et al. 2023) as sister to a clade within the Asgard archaea (Zaremba-Niedzwiedzka et al. 2017; Williams et al. 2020; Liu et al. 2021; Xie et al. 2022; Eme et al. 2023; Zhang et al. 2025). This has provided new evidence for the hypothesis that eukaryotes evolved through a symbiosis between an archaeal and (most likely) one alphaproteobacterial partner (Lopez-Garcia and Moreira 2020; Donoghue et al. 2023; Vosseberg et al. 2024), with the tree of life comprising two primary—the Archaea and Bacteria—and one secondary domain of life—the Eukaryotes (eg Pisani et al. 2007; Williams et al. 2013; McInerney et al. 2014). However, the exact placement of the eukaryotic nuclear and mitochondrial branches remains heavily debated. While several studies have indicated that eukaryotes may have emerged from within the Asgard archaeal class referred to as Heimdallarchaeia, potentially as a sister lineage of the Hodarchaeales (Zaremba-Niedzwiedzka et al. 2017; Williams et al. 2020; Eme et al. 2023), others have suggested that eukaryotes branch sister to the Njordarchaeales (Xie et al. 2022) or the Heimdallarchaeia clade as a whole (Liu et al. 2021; Zhang et al. 2025). Similarly, there is an ongoing debate as to the exact placement of the mitochondria, the powerhouse of eukaryotic cells, with regard to their proteobacterial ancestors. The most recent work implementing complex models of evolution converged on a placement of the mitochondrial branch sister to all core Alphaproteobacteria (Martijn et al. 2018, 2022; Munoz-Gomez et al. 2022), but there is still disagreement in the community (Fan et al. 2020). Accurately placing the eukaryotic mitochondrial and nuclear origins is important as it is essential for reconstructing how the genome of the last common eukaryotic ancestor was assembled, see Kay et al. (2026) for a recent example, thereby informing models of eukaryogenesis (Donoghue et al. 2023). Further improvements in phylogenetic approaches including reconciliation methods that can directly assess contributions from various prokaryotic sources as well as machine learning and structural phylogenetics combined with an ever-better sampling of the vast world of microorganisms and their viruses, promise to yield fascinating new insights into a key transition in cellular evolution that seemed out of reach until so very recently.

The lesson from these examples from two decades of phylogenomics is simple: new data is necessary but not sufficient to ensure progress (Tihelka et al. 2021), which instead relies also on the complementary development of new approaches to more realistically model evolution. Improvements in genome sequencing technologies have been accompanied by advances in high-performance computing, and methodological progress is coming at a spectacular rate (Williams et al. 2021, 2024). Building on the previous work of authors such as Lartillot and Philippe (2004), who introduced infinite mixture models to account for across-site compositional heterogeneity (the CAT-based models) and Foster (2004) who introduced an early model to account for compositional heterogeneity acting across lineages, a diversity of substitution models accounting for the many factors defining the substitution dynamics underpinning molecular evolutionary processes have been developed. Some recent examples include GTRpmix (Banos et al. 2024), allowing the maximum likelihood estimation of exchangeability matrices under a profile mixture model, GHOST (Crotty et al. 2020) to model heterotachy, MAST (Wong et al. 2024) fitting multiple trees to different sets of sites in an alignment, and CAT-PMSF (Szantho et al. 2023) to allow the approximation of CAT-based infinite mixture models in ML settings. With such diversity of models now implemented in scalable maximum likelihood software such as IQTREE (Minh et al. 2020), the possibility to perform novel, insightful research is vast. Some of these approaches have already produced interesting results in the study of notoriously difficult nodes. For example, Giacomelli et al. (2025) recently applied CAT-PMSF to the study of the phylogenetic relationships of the water bears (Tardigrada), while Redmond (2024) used it to test the relationships with Xenoacoelomorpha, an animal lineage including the enigmatic Xenoturbella and the fast-evolving acoels. CAT-PMSF has also found applications in the study of the root of the eukaryotic tree (Williamson et al. 2025) and the placement of Njordarchaeia within the Archaea (Huang et al. 2025). Indeed, CAT-PMSF is a very attractive approach as it combines the strength of both Bayesian and ML approaches (Giacomelli et al. 2025). First, it uses Bayesian software—Phylobayes (Lartillot et al. 2013)—to parametrize an infinite mixture model (either a CAT-Poisson or a CAT-GTR model under a fixed topology). Subsequently, it uses the PMSF (Wang et al. 2018) method to approximate the CAT-based model in IQTREE, where ML analyses are performed, avoiding the challenge to reach convergence frequently encountered with large datasets when, in Bayesian analyses, the parametrization of the CAT-based model and tree search are concomitantly performed. While CAT-PMSF models are approximations of the CAT-Poisson or CAT-GTR models that would be instantiated in Phylobayes, it has been shown by Giacomelli et al. (2025) that CAT-PMSF can fit the data better than both standard across-site compositionally homogeneous models such as WAG (Whelan and Goldman 2001) and empirical profile mixture models (Le et al. 2008).

There is more than sequence data that can be extracted from genome-scale datasets. New data types with phylogenetic potential exist and include, for example, presence and absence of gene families (eg Ryan et al. 2013; Pisani et al. 2015; Leclere et al. 2019; Pett et al. 2019; Juravel et al. 2023; Álvarez-Presas et al. 2024; Schiffer et al. 2024), as well as the more recently developed chromosome fusions with mixing (Simakov et al. 2022). However, new data types should always be evaluated with caution. Phylogeny has a long history of putative “silver bullets” that did not hold up to scrutiny, as presciently pointed out by Hillis (1999), in a paper that remains as relevant today as it was when first published. Chromosome fusions with mixing were initially heralded as one such character type (Schultz et al. 2023), although cautionary notes were presented by Simakov et al. (2022). However, Copley (2025) demonstrated that chromosome fusion events (even if with mixing) are not infallible and should not be considered more reliable than other data types, see also Steenwyk and King (2024). However, when ALGs homology can be confidently established, see Copley (2025) for a discussion, their potential utility is undeniable. This was demonstrated, for example, by Lewin et al. (2025) showing that chromosome fusions provided useful information in the study of the phylogeny of the lophophorates (Brachiopoda, Phronids, and Bryozoa), three animal phyla sharing a specialized filter-feeding structure (the lophophore), the relationships of which proved unstable, that is, model dependent, when addressed using standard sequence data (Laumer et al. 2015; Kocot et al. 2017; Marletaz et al. 2019). However, more studies are necessary to better understand the limits of this data type and how to model it (Steenwyk and King 2024). Other data types such as protein structure-based datasets using 3D structure (Garg and Hochberg 2025; Moi et al. 2025; Mutti et al. 2025) can also be expected to increase in popularity and drive the field forward. Finally, while artificial intelligence approaches in phylogenetics are still in their infancy (Mo et al. 2024), the emergence of transformer-based neural network architecture software such as ChatGPT, and the tremendous impact it had on our daily life, is a powerful reminder that novel AI-based approaches will radically revolutionize our scientific field in ways we cannot predict.

Twenty years of phylogenomics might have led many to think that most problems have been solved and that those that have not been solved yet might be unsolvable. Given the constantly increasing rate at which progress in sequencing and computation has characterized the last two decades, nothing seems further from the truth. We are now placed at a pivotal time in the history of our discipline, with the next decade bound to deliver significant advancements in our understanding of life's origin and diversification. Further biodiversity will be discovered using environmental genomic methods, more and better-quality data will become available and new approaches to analyze the data will continue to be developed. This is not only the time to discover new biodiversity and understand where it fits in the tree of life. Perhaps even more importantly, it is the time to reopen cold cases. It is the time to reinvestigate all those nodes that until yesterday were considered solid, because one thing is certainly true, no phylogenetic result should ever go unquestioned, no matter how robust it might seem, and this is not simply because we have a curiosity about the tree of life. Phylogenetic trees are part of the models we use to understand evolution more broadly. We need accurate phylogenies to infer accurate timetrees (eg Alvarez-Carretero et al. 2022; Carlisle et al. 2023; Mahendrarajah et al. 2023), to reconstruct ancestral morphological phenotypes and understand the evolutionary significance of fossils (eg Rossi et al. 2026). In turn, phylogenies are also used to model processes of genome evolution and reconstruct how ancestral genomes were assembled (eg Paps and Holland 2018; Bowles et al. 2020; Kay et al. 2026), which is key to understand broad processes of adaptation (eg Wei et al. 2026), down to the last universal common ancestor of all cellular life, LUCA (Moody et al. 2024).

Molecular Biology and Evolution has been a powerhouse of molecular phylogenetic research since 1983, and as we enter a period that will be remembered as the golden age of phylogenomics, MBE wants to invite submissions that span all areas of molecular phylogenetic research (both theoretical and applied) across the tree of life (Fig. 1). We look forward to diverse contributions in these pages of MBE in the forthcoming years, with the majority of the papers submitted to this Call of Papers being sent out for review.
